# Lipid peroxidation in patients with vascular mild cognitive impairment from a memory and antioxidants in vascular impairment trial (MOVE‐IT)

**DOI:** 10.1002/alz.095550

**Published:** 2025-01-09

**Authors:** Yejin Kang, Jinghan Jenny Chen, Ethan Mah, Kate Survilla, Danielle Vieira, Nathan Herrmann, Damien Gallagher, Ana C. Andreazza, Sandra E Black, Paul I. Oh, Walter Swardfager, Simon J. Graham, Joel Ramirez, Susan Marzolini, Krista L. Lanctôt

**Affiliations:** ^1^ University of Toronto, Toronto, ON Canada; ^2^ Sunnybrook Research Institute, Toronto, ON Canada; ^3^ Sunnybrook Health Sciences Centre, Toronto, ON Canada; ^4^ Center for Addiction and Mental Health, Toronto, ON Canada; ^5^ KITE Research Institute, Toronto Rehabilitation Institute, Toronto, ON Canada; ^6^ University of Toronto, Torono, ON Canada

## Abstract

**Background:**

Oxidative stress (OS) has been a target of interest for vascular dementia, given its implications in pathogenesis. OS may be important in prodromal stage, such as vascular mild cognitive impairment (vMCI), and examining OS markers in vMCI may help better understand biological processes in the onset of cognitive impairment. Our study compared OS levels in vMCI vs controls, and explored whether OS markers predicted the response to antioxidant treatments in vMCI.

**Method:**

Cardiac rehabilitation patients with cardiovascular risk factors (CVRFs) were recruited as vMCI (1SD below norms in executive function (EF), memory, processing speed or working memory) or cognitively‐healthy controls. vMCI patients were classified as probable vMCI if they had neuroimaging evidence of vascular pathology. vMCI patients were randomized to 2400mg N‐acetylcysteine (NAC) or placebo in a 24‐week, double‐blind trial. Serum 8‐isoprostane (8ISO), 4‐hydroxynonenal (4HNE) and lipid hydroperoxides (LPH) collection and cognitive assessments were done at three time points. ANCOVA models adjusting for age, sex and CVRFs compared OS markers between groups at baseline. Linear mixed‐effects models were used for longitudinal analyses.

**Result:**

LPH levels were significantly lower in vMCI (n = 60) compared to controls (n = 16) (0.86±0.45 vs 1.18±0.31, F(1,62) = 7.0, p = 0.011). 4HNE were significantly higher in vMCI compared to controls (1.03±0.06 vs 0.99±0.04, F(1,70) = 6.6, p = 0.012). vMCI patients had significantly higher 4HNE/LPH (0.17±0.44 vs –0.18±0.29, F(1,62) = 9.3, p = 0.003) and (8ISO+4HNE)/LPH ratio (0.41±0.45 vs 0.13±0.38, F(1,62) = 5.9, p = 0.018) compared to controls. In probable vMCI group (n = 25), there was significant decreases in 4HNE/LPH (β = ‐0.68, SE = 0.27, p = 0.02) and (8ISO+4HNE)/LPH ratio (β = ‐0.65, SE = 0.28, p = 0.028) after NAC treatment. A significant time by baseline 8ISO effect on EF in the NAC group was observed (β = 0.78, SE = 0.26, p<0.01) after adjusting for age, sex and education.

**Conclusion:**

This study showed that the ratios of late‐ to early‐phase lipid peroxidation markers were significantly elevated in vMCI groups compared to controls. This aligns with existing literature that altered lipid peroxidation may be implicated in vMCI. Antioxidant treatments decreased OS in probable vMCI groups, and higher baseline 8ISO was associated with greater improvement in EF in the NAC group, suggesting that probable vMCI patients with high OS may be appropriately targeted for antioxidant treatments.

